# Cognitive and Emotional Irritation in German Veterinarians with Different Levels of Overcommitment

**DOI:** 10.3390/vetsci12040361

**Published:** 2025-04-13

**Authors:** Irina Böckelmann, Emilia Döring, Robert Pohl, Beatrice Thielmann

**Affiliations:** Institute of Occupational Medicine, Faculty of Medicine, Otto von Guericke University Magdeburg, Leipziger Str. 44, 39120 Magdeburg, Germany; irina.boeckelmann@med.ovgu.de (I.B.); emilia.doering@icloud.com (E.D.); robert.pohl@med.ovgu.de (R.P.)

**Keywords:** veterinarians, work engagement, psychological stress, irritation, workload, health promotion, burnout prevention

## Abstract

Background: veterinary medicine is among the most stressful professions and is characterized by long working hours and high emotional demands. This cross-sectional study aimed to investigate the relationship between overcommitment and psychological stress (on the basis of irritation) among veterinarians in Germany, with a particular focus on age differences. Methods: the analysis included a sample of 995 veterinarians divided into three age groups: <35 years, 35–45 years and >45 years. Sociodemographic and job-related data as well as the overcommitment scale (OC, subscale of the Effort–Reward–Imbalance (ERI) questionnaire), and the irritation scale (IS), were both collected. Results: the results reveal that younger (vs. older) veterinarians are significantly more likely to have high overcommitment levels. This group also reported higher-than-average levels of cognitive and emotional irritation, whereas the oldest age group reported comparatively lower overcommitment levels. Age and years of work were negatively correlated with overcommitment, and overcommitment was strongly positively correlated with cognitive and emotional irritation. Conclusions: this study highlights the need for preventative measures to reduce overcommitment and mental stress, particularly among young veterinarians. Interventions during veterinarian studies and in the workplace that promote excessive expectations and stress are crucial to ensure long-term mental health and job satisfaction among this professional group.

## Simple Summary

Veterinary medicine is a highly demanding profession with long working hours and significant emotional strain. This study examined how overcommitment—excessive dedication to work—relates to psychological stress among veterinarians in Germany, particularly across different age groups. A total of 995 veterinarians took part in the study, completing questionnaires about their work habits and stress levels. The results demonstrated that younger veterinarians were more likely to be overcommitted and to experience higher levels of stress, including emotional exhaustion and difficulty disconnecting from work. In contrast, older veterinarians reported lower levels of overcommitment and stress. The study highlights the need for mental health interventions, particularly for younger veterinarians. Stress management training programs and workplace policies that promote work–life balance could help reduce long-term mental health risks. Addressing these challenges is essential to maintaining veterinarians’ well-being and job satisfaction, which ultimately benefits both animal care and public health.

## 1. Introduction

Many people dream of becoming a veterinarian. The opportunity to work with animals daily and promote their health and well-being attracts approximately 1100 young people per year in Germany to study veterinary medicine [1]. A sufficient number of people choose this career path despite the notoriously high demands and stresses of veterinarian study and work—a fact that is highly relevant for safeguarding the health of pets and farm animals in Germany. In a representative survey of German households in 2022, market research institute Skopos counted 34.4 million pets including dogs, cats, small mammals, and ornamental birds. In addition, Germany has numerous ornamental fish and terrarium animals [2]. However, veterinary medicine is responsible not only for the medical care of pets but also for more than 200 million farm animals, such as cattle, pigs, sheep, horses, donkeys, and poultry [3].

Numerous national and international studies have documented the physical and mental workloads associated with working in veterinary medicine and the resulting stresses [4,5,6,7,8,9,10]. In addition to the usual professional challenges, administrative and organizational burdens increase veterinarian stress levels. Veterinary practice owners experience stress resulting from the responsibility of running the practice and competition, whereas employed veterinarians are more likely to report experiencing stress from educational debt, an unclear division of work roles, and low involvement in decision-making processes or work errors [11]. A survey of New Zealand veterinarians provides further evidence of major causes of stress, including working hours, patient owner expectations, unforeseen outcomes, or the need to keep one’s knowledge and skills current. Other stress factors may include social relationships, self-imposed standards, or financial worries [12]. Various studies point to the ethical conflicts and moral stress experienced by veterinarians [13,14,15]. Since veterinarians regularly encounter these conflicts of conscience, they can affect veterinarians’ well-being [13]. Various hurdles—such as the insufficient financial resources of pet owners, which affect the appropriate treatment of animals and therapy options—occur frequently and lead to stress [15]. Less common, but far more stressful, is the pet owner’s request to continue treatments that are sometimes hopeless; euthanasia of healthy animals is almost as stressful [13,15]. Dow et al. found that one-third of veterinarians reported experiencing difficulties in euthanizing animals due to emotional distress or compassion, and 40% reported health problems caused by their interactions with pet owners. Psychological support was not utilized by the veterinarians surveyed [16]. One study revealed that veterinarians who strive for perfection are particularly susceptible to moral stress [14]. Work hours are cited as an important stress factor [17]. Working long hours increases stress levels and poses health risks [5]. With a weekly working time of approximately 50 h, full-time veterinarians (employees and self-employed) work significantly longer than other occupational groups in the German population do [18]. Equine practitioners and practitioners in private clinics have even greater numbers of weekly working hours and see their working of these hours as a source of dissatisfaction [18]. Long working hours are associated with excessive stress and fatigue as well as risky behaviors such as smoking [19].

Another relevant influencing factor is age. Studies suggest that doctors in the younger age group and new entrants to the profession, defined differently in each study, have greater perceptions of stress [11,20,21]. However, studies also demonstrate resources and influencing factors that ensure greater satisfaction among veterinarians [18,22]. These include, for example, good cooperation with colleagues, fair pay, free time and opportunities for relaxation. Respondents who are satisfied with the support they receive from friends and with their work resources experience a resulting positive effect on their resilience [22]. Women in particular stated that family-friendly arrangements were an important workplace feature for them [18].

Compared with the general population, veterinary students suffer more frequently from depression (45.9% vs. 3.2% general population). In addition, the frequency of suicidal thoughts is four times greater among veterinarians (19.9% vs. 4.5% in the general population), and the risk of suicide is approximately three to four times greater (24.0% vs. 6.6% in the general population) [23]. Increased risks of suicide, anxiety and depression in this occupational group can also be found in other studies [6,11,20,24,25,26,27,28]. A scoping review on work-related stress in veterinarians by Pohl et al. examined 28 studies and identified some evidence for a “high prevalence of various risk factors for mental disorders”, including a high risk of suicide and indicators of depression and burnout [7].

When pet owners grieve for their animals, veterinarians can experience severe compassion fatigue and strain. In particular, female and younger veterinarians experience varying degrees of psychological stress [16]. The literature clearly indicates that prospective veterinarians already experience a high level of (health-threatening) stress during their veterinary studies [29,30]. A cross-sectional study on work errors, “adverse events”, and “near misses” revealed that these events had a long-term impact on one third of the veterinarians surveyed in their professional or private lives (e.g., through a loss of confidence in their abilities). Women were more likely to be affected than their male peers were [31]. Clearly, women and younger veterinarians experience high work-related stress levels and poor mental health [12,21,32]. Many studies have identified risk factors in the form of demographic characteristics that affect perceptions of stress or feelings of strain, and the results clearly demonstrate that being female is a risk factor for increased experiences of stress at work. A large proportion of these stress factors are more pronounced among female veterinarians [11,12,32]. This fact is particularly relevant because females in recent decades have increasingly joined the veterinary profession. Approximately three times as many women as men enter the veterinary profession, which may have an impact on well-being in the future [13]. This trend is also observed in data from German veterinary chambers. In terms of the gender distribution across generations, in older age groups (i.e., 60 years and older), the proportion of men significantly exceeds that of women, whereas middle-aged and younger groups (i.e., 59 years and younger) have more female veterinarians [1].

### 1.1. Theoretical Models for Assessing Work-Related Stress and Strain

A number of theoretical models address stress and strain, thereby offering an explanation for the development of work-related experiences of stress and the health impairments caused by workplace stressors. These models include the stress–strain model of Rohmert and Rutenfranz (1975) [33], the effort–reward–imbalance model, and concept of gratification crises by Siegrist (1996) [34], the job demands–resources model of Bakker and Demerouti (2001) [35], the demands–resources model of Becker (2006) [36], and the job–demand–control model by Karasek and Theorell (1990) [37]. This paper uses the effort–reward–imbalance model to better understand the interaction between overcommitment and stress at work and to establish a basis for managing occupational gratification crises. The effort–reward–imbalance model or model of occupational gratification crises describes the reciprocal relationship in professional life between performance (effort) and recognition or reward (Siegrist (1996) [34]). The concept of imbalance arises when efforts and rewards are not equivalent. The concept of gratification crisis is defined as a “violated fairness of exchange between performance and reward”. Siegrist identifies three model components (i.e., central gratification dimensions) that represent recognition (reward) in the work context: “remuneration” (e.g., wages or salary), “career advancement or the granting of job security”, and “nonmaterial forms of recognition and appreciation of achievements” [34]. Work effort can be driven not only by external influences, such as external performance expectations, but also by intrinsic motivation and an “excessive tendency to spend” (overcommitment) in the professional context [34]. People with such overcommitment levels have high work expectations and extend their efforts beyond purely formal requirements. This situation can result in an increased tendency to “frustrate reward expectations” [38]. Frustrations that persist over a longer period of time can lead to stress and health problems. Studies have demonstrated overcommitment among bank employees and an increased risk of burnout as measured by the MBI [39], as well as among anesthetists and intensive care physicians [40]. Again, based on a survey of German employees, Hinsch et al. found an association between critical OC and impaired mental health [41].

The systematic review by Thielmann et al. (2022) revealed that high levels of overcommitment were associated with reduced parasympathetic heart rate variability (HRV) [42]. This suggests an objectively measurable impairment in stress regulation and an increased health risk in overcommitted individuals [42]. This was also similar for teachers. Critical overcommitment in teachers was not only associated with reduced mental health and increased burnout symptoms, but teachers with critical overcommitment also showed reduced HRV [43].

### 1.2. Research Question Development

The previous research summary suggests that veterinarians often experience overload in their work. Mohr and coauthors describe irritation as a form of stress: “Irritation is a state of psychological impairment as a result of experienced goal discrepancy, which includes both rumination (brooding, cognitive irritation), in the sense of increased efforts to achieve goals, and irritability reactions, in the sense of a tendency to defend against goals (emotional irritation)” [44]. This study intends to fulfill the research gap left by the twin phenomena of overcommitment and irritation in the veterinary profession via an age group comparison.

This study focuses on the extent to which overcommitment and work-related stress (irritation) differ among veterinarians in Germany in the age groups studied and how these differences are related to age and professional experience.

It is hypothesized that younger veterinarians (vs. their older colleagues) in the studied sample are more likely to exhibit a high overcommitment levels and above-average levels of cognitive and emotional irritation. An age group comparison is carried out to demonstrate the extent to which the generations differ in these aspects, to obtain a differentiated view of the problem and to derive targeted recommendations and indications for interventions for specific age groups. These findings can be used, for example, in the context of (workplace) health promotion or occupational health practice.

## 2. Materials and Methods

The data come from the cross-sectional study “Causes and consequences of psychological stress in everyday working life and in the emergency service of the veterinary profession in the Federal Republic of Germany”. The survey link, which was active from 1 July 2021 to 31 January 2022, was distributed via state veterinary associations and the German Veterinary Journal. Participation in the study was voluntary, and the survey was anonymous. The full study protocol has been published [45] and is available online (available at https://f1000research.com/articles/11-42, accessed on 9 March 2025). Ethics committee approval is available.

### 2.1. Test Subjects

A total of 995 veterinarians participated in the online survey. The average age was 41.7 ± 10.19 years. Due to incomplete information in the questionnaires used, 163 subjects were excluded from the statistical analyses. The test subjects were divided evenly into three age groups according to their ages (33rd and 66th percentiles): <35 years (AG I), 35–45 years (AG II), and >45 years (AG III).

### 2.2. Questionnaires

Initially, questions were asked about sociodemographics (i.e., age, gender, years worked in the profession, marital status, and household size) and the respondents’ jobs (i.e., employment group, place of work, job status, specialty, chamber affiliation, and employment contract). Standardized questionnaires were used to record the effects of psychological stress on those working in the veterinary profession: the overcommitment scale (OC), which is a subscale of the effort–reward–imbalance questionnaire [34], and the irritation scale [44].

#### 2.2.1. Overcommitment Scales

The overcommitment (OC) scale is a subscale of the effort–reward–imbalance (ERI) questionnaire [34,46]. The ERI is used to measure occupational gratification crises for psychosocial risk assessments. It measures the relationship between effort and reward at work and the tendency to overcommit [38]. The questions on overcommitment, i.e., an excessive tendency to expend effort, are relevant for this study. The possible answers on a four-point Likert scale are “strongly disagree”, “somewhat disagree”, “somewhat agree”, and “strongly agree” [46]. This setup results in a total score of 6–24 points for overcommitment values. As the subscale defines no standard values, this evaluation primarily considers the data distribution [46]. For this study, quartiles were formed from the sum score of the OC scale. As the sum score of the fourth quartile is assessed as a critical overcommitment, an overcommitment value of 18 points or more in the examined sample is considered a “critical” overcommitment. Many prospective epidemiological studies support this OC classification [39,43].

Various cutoff values for overcommitment levels are used in the literature to classify overcommitment levels as “high” and to designate the respondent correspondingly as overcommitted [39,43,46,47,48,49,50]. Data on Cronbach’s α from various publications on the ERI indicate good reliability. The Cronbach’s α, which is a measure of internal consistency, is most often >0.70 [46,51,52].

#### 2.2.2. Irritation Scale

The irritation scale (IS) was used to record the consequences of work-related stress [44,53]. Cognitive irritation (CI) and emotional irritation (EI) are two subscales of the questionnaire. Cognitive irritation is associated with workload and job characteristics and thus involves continuing to address work-related problems outside of working hours or during leisure time. The term “work-related rumination” is also frequently used in the context of constantly recurring thoughts about work. Emotional irritation, on the other hand, provides indications of social stressors. Emotional irritation can manifest itself, for example, in irritability and verbally aggressive behavior. These are the two ways in which stress can be experienced. Fundamental to the concept of irritation is that the stress disorder is caused by work-related factors [44]. The application area extends to occupational health analyses, evaluations of health interventions, therapeutic practice, and research.

The respondents were asked to indicate the extent to which they agreed with the eight items, presented as statements on a 7-point scale: “strongly disagree”, “mostly disagree”, “slightly agree”, “moderately agree”, “somewhat agree”, “mostly agree”, and “almost completely agree” [44]. Items 3, 5, 6, 7, and 8 describe the emotional irritation factor, whereas Items 1, 2, and 4 ask about cognitive irritation aspects. The evaluation can be carried out either as an overall index (total raw scores from 7 to 56) or via the two subscales (sum of the values of the emotional irritation items from 5 to 35 points or, in the case of cognitive irritation, from 3 to 21 points). These data can then be converted into norm values, so they can be compared with the response behavior of the norm sample. The norm values are measured in percentiles and stanine values. Percentile norms are used because the data are not normally distributed in the survey with the irritation scale (measured on a norm sample). The stanine values for emotional and cognitive irritation as well as the overall irritation index (GI) are as follows: “below average” (stanine values 1–2), “average/normal” (stanine values 3–7), and “above average” (8–9). The survey instrument fulfills the quality criteria of reliability, validity, and objectivity. The Cronbach’s α, measured on 15 samples, is between 0.85 and 0.93 [53].

### 2.3. Statistical Procedures

IBM SPSS Statistics Version 28 (Armonk, NY, USA) was used for the statistical analyses. The data were tested for a normal distribution via the Kolmogorov–Smirnov test. After frequency analyses were performed, the percentiles were calculated at 33% and 66% for the age and overcommitment variables, respectively. Cross-tabulations with Pearson’s chi-square tests were created, and the tau test (Goodman and Kruskal) was used to analyze the subgroup distribution in the age groups.

Descriptive statistics were used to calculate the mean values, standard deviations, medians, minimums, maximums, and 95% confidence intervals in the three defined age groups. The Kruskal–Wallis test was used to compare the differences between independent groups. The significance level was a 5% probability of error. The Bonferroni correction was also applied for multiple comparisons.

In addition, generalized linear models (GLMs) were used to analyze the multivariate effects of the independent variables on the dependent variables. The *p* value and the partial eta square, which reflects the strength of the effect of the independent variable, were determined. An eta square of 0.01 corresponds to a small effect, 0.06 corresponds to a medium effect, and a large effect is indicated by 0.14 [54]. In this study, the eta-squared value is also given as a percentage to illustrate the explanatory power of the independent variable more clearly.

As the data were not normally distributed, nonparametric correlation analyses were performed according to Spearman’s test, and two-sided significance was tested. To describe the magnitude of the correlation coefficient (rho or ρ), gradations were defined according to Cohen 1988 [54].

## 3. Results

### 3.1. Sociodemographic and Occupational Data

The total sample size was 995 subjects between the age of 23 and 79, 650 of whom were female (65.3%). The average age was 41.7 ± 10.19 years (Table 1). There was a statistically significant difference between the age groups for the age variable.

Each of the three age groups comprised approximately one third of all test subjects (division of the total sample according to the 33rd and 66th percentiles): 33.4% (n = 332) belonged to AG I (<35 years), 34.1% (n = 339) were AG II (35–45 years), and 32.6% (n = 324) were AG III (>45 years) (Table 1). Overall, 71.1% of the participants in Age Group I were female (Table 2); this proportion was smaller in the other age groups (67.0% women in AG II and 57.7% in AG III).

Fifty-nine percent of all respondents stated that they were single (Table 2). A further 35% were married. A total of 1.1% were widowed, and 4.6% were divorced. There were significant differences between the age groups in terms of sex and marital status (*p*_χ_^2^ < 0.001). The people in AG I were more likely to be single (76.2%) than were those in AG II (58.1%) and AG III (42.3%). The converse was true for the number of people who were married. Of AG I, 23.5% were married. A total of 37.2% were in AG II, and 45.4% were in the oldest age group. This result indicates that in the studied sample, older veterinarians were more frequently married, and younger veterinarians had a greater tendency to be single.

When asked whether the respondents had children in their household, 746 people answered. A total of 53.9% stated that they had children in the household. A total of 46.1% answered negatively (Table 2). In AG I, 77.22% (n = 193) of the participants answered “yes”. In AG II, 51.4% (n = 126) of the participants in the age group had children in the household. The lowest proportion was in AG III. A total of 33.1% (n = 83) of the participants had children in the household. There was a significant difference between the age groups (*p*_χ_^2^ < 0.001). The number of years employed at the time of the survey averaged 14.2 ± 9.95 years (Table 1).

Table 3 shows the employment group distribution within the overall sample and the age groups. A total of 40.3% (n = 401) stated that they were self-employed/practitioners. A total of 25.3% were trainee or assistant doctors. The third largest group, at 15.9%, were employees of a practice or clinic. Within the youngest age group, 16% were self-employed, 28% were employed in practices or clinics, and 38.9% were trainee or assistant doctors. AG II had considerably more self-employed individuals and practitioners than did the other groups, with a share of 38.1%. The proportion of employees in practices and clinics was lower than that in AG I and amounted to 13.6%. The situation was similar for trainees and assistant doctors (3.2%). In the oldest age group, the proportion of self-employed people was highest at 67.3%. At the same time, AG III had the lowest proportion of employees in practices and clinics (5.9%). Trainee and assistant doctors comprised 10.5% of the respondents in AG III, which was greater than that in AG II but considerably less than that in AG I. The age groups differed significantly regarding the employment groups (*p*_χ_^2^ 2 < 0.001).

When the distributions in the specialist areas were analyzed, the majority of the test subjects (55.1%, n = 548) were found to specialize in small animals (Table 4). Regarding the age groups, a statistically significant difference was found in the specialty distribution (*p*_χ_^2^ < 0.001). The proportions of small animal veterinarians in the three age groups were roughly identical. Large animal veterinarians (specializing in livestock and horses) accounted for the second largest proportion. A total of 17.4% of all respondents (n = 173) worked in this specialty. However, 21.7% of the youngest age group specialized in large animals, and 19.2% of the middle-aged group did so. The lowest proportion of large animal veterinarians was recorded in AG III, with 11.1%. A total of 15.1% (n = 150) of the veterinarians surveyed treated both small and large animals. A total of 34.7% of these were in AG I. An additional 29.3% of the small and large animal veterinarians were in the middle-aged group, and the remaining 36.0% were in the older age group. Only very few younger veterinarians worked for public authorities (3% in AG I vs. approximately 12% in AG II and III).

The survey also asked whether the veterinarians surveyed worked in a large city, in a medium-sized or small town (under 100,000 inhabitants), or in a rural area. A total of 268 respondents indicated a large city as their place of work, which corresponds to 26.9% of the respondents (Table 4). With respect to the age groups, younger veterinarians may work more frequently (30.7%) in a large city. However, the differences between the age groups were not significant in terms of workplace (*p*_χ_^2^ = 0.352).

Only 980 people answered the question about their employment contract. Of these, 578 (59%) stated that they had an open-ended employment contract (Table 4). In AG I, one fifth (20.5%) had a fixed-term contract. In contrast, this figure was 4.5% for AG II and only 1.9% for AG III. In AG I, 71.0% stated that they had an open-ended contract; in the middle-aged group, it was 67.2%; and in the older age group, it was 37.9%. The selection category “does not” means that the respondent was likely self-employed. This percentage increased as the age groups increased (AG I: 8.5%, AG II: 28.3%, and AG III: 60.3%), which is a clear indication of increasing self-employment with increasing age. Differences were found in the age groups regarding the employment contract distribution (*p* < 0.001).

### 3.2. Overcommitment

For the overall sample, the OC sum score had a mean value of 16.0 ± 3.10 points (Table 5). In AG I, the overcommitment value was slightly higher at 16.3 ± 3.14 points. This result means that the youngest age group was below the cutoff value of 18 points on average and therefore had a normal tendency to overcommit. The mean value was 16.3 ± 3.05 points, with AG II showing very similar values. Older veterinarians again reported lower overcommitment values (15.6 ± 3.06 points). The three age groups differed significantly from each other in terms of overcommitment (*p*_Kruskal–Wallis_ = 0.002). In the subsequent analysis of the age groups, significant differences were found between the youngest and oldest age groups after Bonferroni correction (AG I vs. AG II: *p*_Bonferroni_ = 0.004), and between the middle and oldest age groups (AG II vs. AG III: *p*_Bonferroni_ = 0.007).

Of the 995 test subjects, 335 achieved a total overcommitment value greater than 18 points (Table 6). This result means that 33.7% of the sample had critical values for their occupational overcommitment levels. The highest proportion of people with increased overcommitment values (36.9%) was among veterinarians in AG II. AG I was slightly lower, with a share of 36.1%. The lowest proportion of subjects with critical overcommitment values was found in AG III (27.8%). These differences were statistically significant (*p*_χ_^2^ = 0.023). From the data obtained, an overall high overcommitment level among young veterinarians can be deducted.

### 3.3. Irritation as Stress

In terms of cognitive irritation, a value of 7.99 ± 1.390 stanine points was recorded for the overall sample, indicating above-average stress levels (Table 5). The analysis of emotional irritation revealed stanine values of 7.50 ± 1.531 points, which also indicates above-average emotional stress levels among the veterinarians surveyed. Accordingly, the stanine values for the overall index were above the reference range at 7.80 ± 1.409 points. The different age groups significantly differed in all the irritation scales (i.e., cognitive, emotional, and total index irritation) (*p*_Kruskal–Wallis_ < 0.001). The younger age group presented the greatest degree of cognitive irritation (AG I vs. AG III *p*_Bonferroni_ < 0.001). AG II also exhibited significantly greater cognitive irritation than did AG III (*p*_Bonferroni_ < 0.001). AG II had higher values for emotional irritation. The older age groups also exhibited more favorable values on the overall index than the other two age groups did (*p*_Bonferroni_ < 0.001).

More than half of the samples had above-average values on the stanine scale regarding emotional irritation (56.1%). The remaining 43.9% had irritation levels that were within the normal range (stanine values 3–7) (Table 6). Overall, the stanine values for emotional irritation in the studied sample were well below those for cognitive irritation (55.7% of AG I). Sixty-four percent of AG II and 48.1% of AG III achieved above-average stanine values regarding their emotional irritation levels. As with cognitive irritation, significant group differences were noted in terms of emotional irritation (*p*ρ 2 according to Pearson < 0.001).

Regarding cognitive irritation, 280 people (28.1%) achieved average stanine values, and 715 people had above-average stanine values (71.9%). In AG I, 77.4% of the participants had an above-average score for cognitive irritation (Table 6). In AG II, this percentage was 75.2%. The oldest age group, on the other hand, had the lowest level of cognitive irritation, at 62.7% (*p*ρ 2 according to Pearson < 0.001).

For the surveyed veterinarians, sex, employment type, specialist area, working years, and age group offered no explanatory power in the irritation context (Table 7). In contrast, specialty partially explained the differences found in cognitive irritation (η^2^ = 0.007 at *p* = 0.027). Overcommitment can be seen as a predictor with a small effect and explained the dependent variables CI by 3.8% (*p* < 0.001), EI by 5.1% (*p* < 0.001), and TI by 4.9% (*p* < 0.001).

The nonparametric correlations according to Spearman’s rho are shown in Table 8. The age of the test subjects correlated positively with the number of years worked in the profession (ρ = 0.931 at <0.001). In addition, the results indicated that older subjects who have worked more years in the profession had lower irritation scores. This result was true for AI and years worked (ρ = −0.158; *p* < 0.001), CI and age (ρ = −0.145; *p* < 0.001), EI and years worked (ρ = −0.086; *p* = 0.007), and EI and age (ρ = −0.082; *p* = 0.010). Similarly, the total index of irritation correlated significantly with age (ρ = −0.116; *p* < 0.001) and years worked in the occupation (ρ = −0.123; *p* < 0.001). Overcommitment was significantly negatively related to age (ρ = −0.099; *p* 0.002) and years worked (ρ = −0.104; *p* = 0.002), i.e., in the studied sample, older veterinarians and those who had been in the profession for many years tended to be less inclined to overcommit.

## 4. Discussion

This cross-sectional study aimed to investigate the relationships between overcommitment and the consequences of stress in veterinarians, considering the differences among three age groups. The sample is representative in terms of gender distribution. In Germany, the overall proportion of women in veterinary medicine is 65.7%, and 65.3% of the studied sample also consisted of female veterinarians. To better compare the ages of the sampled subjects with the veterinary statistics of the German Veterinary Association (2023), the same age groups were compared [1]. The proportion of older veterinarians was greater in the population than it was in the studied sample (20.2% of the doctors in the veterinary statistics of the German Veterinary Association are 40–49 years old, 21.0% are 50–59 years old, 16.7% are 60–69 years old, and 8.4% are over 69 years old). Only 5.3% of the veterinarians surveyed in the study were aged 60 and over. In the age group 50–59 years old, the proportion of veterinarians in the studied sample (20.0%) was comparable to the proportion of veterinarians of the same age in the population (21.0%). The proportion of surveyed veterinarians aged 40–49 years was 27.7%. These statistics indicate that the studied sample is less representative overall, as it is somewhat younger.

In this manuscript, overcommitment is addressed as a key psychological risk factor that is closely associated with several health outcomes. As outlined in the introduction, numerous studies have demonstrated associations between high levels of occupational overcommitment and negative health outcomes, including burnout, reduced heart rate variability (HRV), depressive symptoms, and even increased suicidality (e.g., [6,7,42,46,55]). The discussion deliberately revisits these aspects in order to critically contextualize the empirical findings of the current sample—in particular the above-average levels of overwork and irritability observed among younger vets—and to relate them to the existing body of evidence. In this context, the stresses identified are not just professional challenges but may pose serious threats to mental health, highlighting the urgent need for preventive action.

As hypothesized, indications were obtained of the consequences of stress in the form of cognitive and emotional irritation and excessive willingness to overcommit, and many correlations were identified. The younger veterinarians and those just starting out in their careers were often characterized by high overcommitment levels. The proportion of veterinarians with an excessive tendency to overcommit (approximately 1/3 in each age group) was statistically comparable between the two younger samples, although the proportion of veterinarians with critical overcommitment was lower among older veterinarians (approximately 1/4). In terms of their OC sum values, the veterinarians in the youngest age group differed significantly from those in the oldest age group. On average, the entire sample and each age group presented high stress levels. Both cognitive and emotional irritation were above the reference ranges. In terms of cognitive irritation, this result was particularly pronounced in age group I. The proportion of veterinarians with an above-average level of emotional irritation was well over half of all young veterinarians and was even greater among middle-aged veterinarians.

In an exploratory study, employees of the Norwegian Air Rescue Service were asked which factors can prevent excessive commitment, i.e., overcommitment [56]. The most frequently mentioned factors included planning for emergencies, communication, quality and flow of information, training, and preparation and teamwork behavior. However, work experience was also frequently mentioned by the test subjects.

Among the surveyed veterinarians, age was strongly correlated with years of work (in the profession). A cross-sectional study on overcommitment among nurses also revealed correlations between professional experience and age, on the one hand, and overcommitment, on the other hand. People with 10 years or more of work experience had a lower risk of developing high overcommitment levels. The same applied to nurses who were 30 to 40 years old or more than 40 years old [57]. Various studies in other occupational groups have provided evidence of the harmful effects of high overcommitment levels on health. In a systematic review by Thielmann et al. (2022), people with high overcommitment values presented reduced parasympathetic HRV parameters [42]. High OC levels are associated with the occurrence of cardiovascular diseases and depressive disorders [46,55]. Du Prel et al. (2015) examined overcommitment in connection with the inability to work [58]. People with high OC levels were more frequently unable to work for up to 42 days as well as for long periods. Overcommitment was also investigated in kindergarten teachers. A high overcommitment level was negatively correlated with the subjective assessment of mental health. Furthermore, indications were obtained of the connection between overcommitment and HRV [43]. A study from Taiwan demonstrated that a high overcommitment level in civil servants was associated with a significant deterioration in mental and physical health [59]. Compared with their older colleagues (AG III), the younger veterinarians (AG I) in the studied sample presented a greater overcommitment level and above-average cognitive and emotional irritation levels. Other studies have revealed that younger veterinarians in particular report higher stress levels and poorer mental health [16,21]. The correlation between overcommitment and the irritation level observed in the sample was also confirmed in a further cross-sectional study of students who work during their studies [60]. Strong positive correlations were found between overcommitment values and the two irritation subscales. For veterinarians, the correlation between overcommitment and cognitive irritation was stronger than was that between overcommitment and emotional irritation.

Various studies have also found correlations between overcommitment and other scales that were not surveyed in this way among veterinarians. One example is the quality-of-life factor. In a cross-sectional study, high overcommitment levels among Brazilian nurses correlated with a lower quality of life [49]. High overcommitment levels have been negatively correlated with self-perceived mental health [41] and the risk of burnout among bank employees [39] and Vietnamese medical professionals [61]. Furthermore, overcommitment and burnout were examined in a cohort of U.S. police officers. Overcommitment was positively related to the burnout subscales of cynicism and exhaustion. Additionally, a high overcommitment score was associated with poorer job performance [62]. Not all of the correlations found between overcommitment and irritation can be confirmed by studies. Nevertheless, a large proportion of the findings is reflected in the current literature. Thus, in connection with the results, a uniform picture emerges, demonstrating a need for action for (occupational) health promotion and occupational medicine as a preventive medical discipline.

### 4.1. Recommendations

One relevant starting point is the study of veterinary medicine. In the best-case scenario, students should be adequately prepared for the stresses of the profession and be sensitized to maintaining their mental health. Practice areas include communication with animal owners, administration, and time management [10], which should be included in the curriculum to minimize mental stress and illnesses caused by the profession or its study [26].

Bahramsoltani et al. compared various communication courses in an intervention study [63]. Veterinary students who completed a practice-oriented course, including role play and real-life situations, rated their communication skills better than did those who completed an e-learning program or did not attend any of the courses. However, the participants were not blinded in the study [63]. Nevertheless, the data demonstrate the relevance of practical implementation in veterinary studies. In general, little research to date has been conducted on (effective) interventions that are specifically tailored to the veterinary profession. In a Scottish cross-sectional study on ethical dilemmas, 78% of the surveyed veterinarians stated that their studies had inadequately prepared them for the ethical aspects of their work [13]. This finding could also apply to German veterinarians, as BGW rates making “quick decisions in critical situations” and the topic of euthanasia in its “risk assessment in veterinary medicine” as psychologically stressful [64]. Therefore, this would be another important point that should be included in the curriculum alongside medical content. Involving veterinary universities and faculties as prevention actors makes sense, particularly because younger veterinarians show psychological stress consequences (frequent above-average irritation with higher overcommitment values). At the same time, veterinary students must already cope with a high workload regarding their courses, examinations, etc., and an additional load such as that represented by required workshops on stress management should be integrated in such a way that they do not represent an additional burden for the students. In addition to universities, the Bundesverband Praktizierender Tierärzte e. V. is a relevant stakeholder. It can represent the professional interests of veterinarians vis-à-vis political institutions, authorities and other organizations [65], specifically, via lobbying and cooperation with political decision-makers. According to a British study, working veterinarians would like to have more influence on working hours and the opportunity to change management and team aspects [66], but calls have also been made among German veterinarians for measures to limit working hours and evening shifts [10]. Professional associations such as the Bundesverband Praktizierender Tierärzte e. V. can work with policymakers to promote legislation on working hours and shifts that meet the needs of veterinarians. A survey on the job satisfaction of employed veterinarians in Germany revealed that those who worked fewer night and weekend shifts were more satisfied. Nevertheless, comprehensive medical and nursing care for pets and farm animals must be guaranteed, which could make it difficult to implement family-friendly working hours, particularly in regions with a low density of veterinarians.

Stress management and health promotion topics can be integrated into training courses to minimize stress caused by long working hours and shift work, ethical dilemmas or difficult discussions with patients. Workshops and seminars on practice management and time management can be promising for training practice owners or people in management positions in clinics on how to implement more efficient and employee-friendly working time models. The DVG (Deutsche Veterinärmedizinische Gesellschaft) promotes scientific research and continuing education in veterinary medicine and could act as a partner for workshops and prevention programs [67]. Batchelor and McKeegan noted the need for ongoing training for practicing veterinarians, as the topic of ethics in particular has not always been part of the curriculum [13]. In general, further training in medical professions is highly valued and occurs regularly, offering the opportunity to integrate health promotion content. As the state veterinary chambers are also responsible for the professional supervision and further training of veterinarians, it makes sense to involve the chambers in any project. Finally, the careful implementation of a legally required risk assessment is an instrument that can help to provide the impetus for concrete measures. A regular evaluation of the measures and their adjustment on the basis of feedback are relevant to ensure the effectiveness of the programs and their continual further development. General reference should always be made to good practice approaches so that health promotion can be carried out on the basis of evidence and with the appropriate quality of work.

The recommendations for preventive measures in the veterinary context, as expressed in the manuscript, are based on the identified results and general findings from related professions. It should be emphasized, however, that many of these recommendations should be viewed with a degree of caution due to the lack of robust empirical studies and evidence-based interventions specific to veterinary practice—a circumstance that is not uncommon in preventive settings. However, this should not be seen as an argument against developing or applying appropriate interventions. Rather, we advocate a critical but open-minded examination of preventive approaches, which, in the best case, can be evaluated and developed on the basis of evidence.

### 4.2. Limitations

One limitation of this research is the cross-sectional design. Such a design aids in the identification of correlations but not causalities. As the subjects assessed themselves on the selected survey instruments, their subjective perceptions were recorded, not objective parameters. Response bias due to social desirability cannot be ruled out either. Participation was only possible online, which led to a biased selection of test subjects with regard to the gender distribution in the age groups. Older test subjects in particular were less frequently represented in the studied sample and were therefore underrepresented. This limitation affects the external validity of age-related conclusions. This result could be due to the lower affinity of older generations for digital technologies and the internet. Therefore, future studies should consider alternative survey methods to ensure a more balanced age distribution. As the survey was conducted online, there is a tendency to attract people with a higher digital affinity, which may inadvertently exclude potential participants who are less technologically inclined—particularly older people and those living in rural areas.

As demonstrated in the Discussion, the results can be compared with those of other occupational groups, but a comparison within the occupational group is not possible owing to the lack of corresponding comparative data from studies.

Regarding the evaluation of the irritation scale, standard values, which are now outdated, were used at the time of the study. More recent studies should use the standard values from Gralla et al. 2023 [68].

## 5. Conclusions

The data obtained from the age group comparisons can be used for future research projects to focus interventions on young veterinarians and possibly middle-aged veterinarians. Future research could focus on developing effective interventions to reduce overcommitment and irritation and evaluating their effectiveness. In addition, longitudinal studies could provide deeper insight into the development of stress and its impact on the long-term mental and physical health of veterinarians. The influence of demographic factors such as gender, specialty and the work environment on stress and resilience should also be further investigated.

The results of this study not only provide important impetus for the scientific debate on mental stress in veterinary medicine but also highlight the need for a coordinated approach by educational institutions, professional associations, and political decision-makers. Sustainable solutions that promote both the job satisfaction and health of veterinarians in the long term can be developed only through the joint efforts of all stakeholders.

## Figures and Tables

**Table 1:** Age and years in the profession of the total sample and the three groups.

Variable	Total (n = 995)	Age Groups			*p*_Kruskal–_ _Wallis_	*p*_Bonferroni_
		AGI (n = 332; 33.4%)	AGII (n = 339; 34.1%)	AGIII (n = 324; 32.6%)		
		MW ± SD Median (Min-Max) [95%CI]				
**Age** **[years]**	41.7 ± 10.19	31.0 ± 3.02	40.4 ± 3.02	54.0 ± 5.49	<0.001	I-II (<0.001)
		31 (23–35)	40 (36–45)	54 (46–79)		I-III (<0.001
		[30.69–31.34]	[40.13–40.72]	[53.44–54.64]		II-III (<0.001)
**Professional experience [years]**	14.2 ± 9.95	4.70 ± 2.799	12.7 ± 4.79	25.6 ± 6.82	<0.001	I-II (<0.001
		5 (1–11)	13 (1–45)	25 (6–50)		I-III (<0.001)
		[4.40–5.00]	[12.20–13.22]	[24.88–26.37]		II-III (<0.001)

**Table 2:** Distribution of gender, marital status, and children in the household within the overall sample and within the three age groups (number (%)).

Variable	Total (n = 995)	Age Groups		
		AG I (n = 332)	AG II (n = 339)	AG III (n = 324)
**Gender**				
*Male*	345	96	112	137
% of age group	34.7%	28.9%	33.0%	42.3%
% of the total number	34.7%	9.6%	11.3%	13.8%
*female*	650	236	227	187
% of age group	65.3%	71.1%	67.0%	57.7%
% of the total number	65.3%	23.7%	22.8%	18.8%
Note: *p*_χ2 according to Pearson_ = < 0.001; *p*_Goodman-and-Kruskal-Tau_ < 0.001 (age-group-dependent)				
**Marital status**				
*single*	587	253	197	137
% of age group	59%	76.2%	58.1%	42.3%
% of the total number	59%	25.4%	19.8%	13.8%
*married*	351	78	126	147
% of age group	35.3%	23.5%	37.2%	45.4%
% of the total number	35.3%	7.8%	12.7%	14.8%
*widowed*	11	1	2	8
% of age group	1.1%	0.3%	0.6%	2.5%
% of the total number	1.1%	0.1%	0.2%	0.8%
*divorced*	46	0	14	32
% of age group	4.6%	0%	4.1%	9.9%
% of the total number	4.6%	0%	1.4%	3.2%
Note: *p*_χ2 according to Pearson_ < 0.001; *p*_Goodman-and-Kruskal-Tau_ < 0.001 (age-group-dependent)				
**Participants who have children**				
Quantity	746	250	245	251
**Participants who have children in the household (excluding childless participants)**				
*No*	344	57	119	168
% of age group	46.1%	22.8%	48.6%	66.9%
% of the total number	46.1%	7.6%	16.0%	22.5%
*Yes*	402	193	126	83
% of age group	53.9%	77.2%	51.4%	33.1%
% of the total number	53.9%	25.9%	16.9%	11.1%
Note: *p*_χ2 according to Pearson_ = < 0.001; *p*_Goodman-and-Kruskal-Tau_ < 0.001 (age-group-dependent)				

**Table 3:** Distribution of employment groups within the overall sample and within the three age groups (number (%).

Employment	Total (n = 995)	Age Groups		
		AG I (n = 332)	AG II (n = 339)	AG III (n = 324)
*Self-employed/practitioner*	401	54	129	218
% of age group	40.3%	16.3%	38.1%	67.3%
% of the total number	40.3%	5.4%	13.0%	21.9%
*Employed in the public sector*	98	30	41	27
% of age group	9.8%	9.0%	12.1%	8.3%
% of the total number	9.8%	3.0%	4.1%	2.7%
*Civil servants*	32	0	15	17
% of age group	3.2%	0%	4.4%	5.2%
% of the total number	3.2%	0%	1.5%	1.7%
*Private sector/industry*	30	13	11	6
% of age group	3.0%	3.9%	3.2%	1.9%
% of the total number	3.0%	1.3%	1.1%	0.6%
*Trainee/resident doctors*	252	129	89	34
% of age group	25.3%	38.9%	3.2%	10.5%
% of the total number	25.3%	13.0%	8.9%	3.4%
*Other activity*	15	5	8	2
% of age group	1.5%	1.5%	2.4%	0.6%
% of the total number	1.5%	0.5%	0.8%	0.2%
*Without practicing*	1	0	0	1
% of age group	0.1%	0%	0%	0.3%
% of the total number	0.1%	0%	0%	0.1%
*PhD students*	8	8	0	0
% of age group	0.8%	2.4%	0%	0%
% of the total number	0.8%	0.8%	0%	0%
*Employed in practice/clinic*	158	93	46	19
% of age group	15.9%	28.0%	13.6%	5.9%
% of the total number	15.9%	9.3%	4.6%	1.9%
Note: *p*_χ2 according to Pearson_ < 0.001; *p*_Goodman-and-Kruskal-Tau_ < 0.001 (age-group-dependent)				

**Table 4:** Distribution of specialist areas by type of animal, place of work, and type of employment contract within the overall sample and within the three age groups (number (%)).

	Total (n = 995)	Age Groups		
		AG I (n = 332)	AG II (n = 339)	AG III (n = 324)
**Department**				
*Small animals*	548	184	179	185
% of age group	55.1%	55.4%	52.8%	57.1%
% of the total number	55.1%	18.5%	18%	18.6%
*Large animals (livestock and horses)*	173	72	65	36
% of age group	17.4%	21.7%	19.2%	11.1%
% of the total number	17.4%	7.2%	6.5%	3.6%
*Small and large animals*	150	52	44	54
% of age group	15.1%	21.7%	13.0%	16.7%
% of the total number	15.1%	5.2%	4.4%	5.4%
*Laboratory area*	34	14	11	9
% of age group	3.4%	4.2%	3.2%	2.8%
% of the total number	3.4%	1.4%	1.1%	0.9%
*Authority*	90	10	40	40
% of age group	9.0%	3.0%	11.8%	12.3%
% of the total number	9.0%	1.0%	4.0%	4.0%
Note: *p*_χ2 according to Pearson_ < 0.001; *p*_Goodman-and-Kruskal-Tau_ < 0.001 (age-group-dependent)				
**Place of work**				
*City* *(> than 100,000 inhabitants)*	268	102	80	86
% of age group	26.9%	30.7%	23.6%	26.5%
% of the total number	26.9%	10.3%	8%	8.6%
*Medium/small town* *(< 100,000 inhabitants)*	334	107	119	108
% of age group	33.6%	32.2%	35.1%	33.3%
% of the total number	33.6%	10.8%	12.0%	10.9%
*Rural area*	393	123	140	130
% of age group	39.5%	37.0%	41.3%	40.1%
% of the total number	39.5%	12.4%	14.1%	13.1%
Note: *p*_χ2 according to Pearson_ = 0.352; *p*_Goodman-and-Kruskal-Tau_ = 0.347 (age-group-dependent)				
**Employment contract is...**	**(n = 980)**	**(n = 331)**	**(n = 332)**	**(n = 317)**
*Temporary*	89	68	15	6
% of age group	9.1%	20.5%	4.5%	1.9%
% of the total number	9.1%	6.9%	1.5%	0.6%
*Unlimited*	578	235	223	120
% of age group	59.0%	71.0%	67.2%	37.9%
% of the total number	59.0%	24.0%	22.8%	12.2%
*Does not apply*	313	28	94	191
% of age group	31.9%	8.5%	28.3%	60.3%
% of the total number	31.9%	2.9%	9.6%	19.5%
Note: *p*_χ2 according to Pearson_ < 0.001; *p*_Goodman-and-Kruskal-Tau_ < 0.001 (age-group-dependent)				

**Table 5:** Overcommitment and irritation scales within the overall sample and within the three age groups.

Variable		Age Groups			*p*_Kruskal–Wallis_	*p*_Bonferroni_
	Total (n = 995)	AGI (n = 332)	AGII (n = 339)	AGIII (n = 324)		
		MW ± SD Median(Min-Max) [95%CI]				
**Overcommitment**						
OC: Total score of all items	16.0 ± 3.10	16.3 ± 3.14	16.3 ± 3.05	15.6 ± 3.06	0.002	I–III (0.004) II–III (0.007)
		17 (6–24)	16 (6–24)	15.5 (6–24)		
		[15.91–16.59]	[15.93–16.58]	[15.26–15.93]		
**Irritation**						
Cognitive irritation (sum score)	15.02 ± 4.865	15.79 ± 4.640	15.37 ± 4.565	13.87 ± 5.186	<0.001	I–II (0.001) I–III (<0.001)
		17 (3–21)	17 (3–21)	15 (3–21)		
		[15.29–16.29]	[14.88–15.85]	[13.30–14.44]		
Cognitive irritation (stanine value)	7.99 ± 1.390	8.91 ± 1.298	8.11 ± 1.274	7.65 ± 1.534	<0.001	I–III (<0.001) II–III (<0.001)
		9 (4–9)	9 (4–9)	8 (4–9)		
		[8.05–8.33]	[7.97–8.24]	[7.48–7.82]		
Emotional irritation (sum score)	19.13 ± 7.804	19.41 ± 7.706	20.09 ± 7.503	17.82 ± 8.056	<0.001	I–III (0.024) II–III (0.001)
		19 (5–35)	20 (5–35)	17 (5–35)		
		[18.58–20.24]	[19.29–20.89]	[16.94–18.70]		
Emotional irritation (stanine value)	7.50 ± 1.531	7.57 ± 1.484	7.71 ± 1.450	7.20 ± 1.618	<0.001	I–III (<0.001) II–III (<0.001)
		8 (4–9)	8 (4–9)	7 (4–9)		
		[7.41–7.73]	[7.55–7.86]	[7.02–7.38]		
Total index	34.15 ± 11.468	35.20 ± 11.116	35.45 ± 10.899	31.69 ± 12.034	<0.001	I–III (<0.001) II–III (<0.001)
		36 (8–56)	36 (10–56)	32 (8–56)		
		[34.00–36.40]	[34.29–36.62]	[30.38–33.01]		
Total index (stanine value)	7.80 ± 1.409	7.92 ± 1.325	8.00 ± 1.279	7.48 ± 1.563	<0.001	I–III (0.001) II–III (<0.001)
		8 (3–9)	8 (4–9)	8 (3–9)		
		[7.78–8.06]	[7.86–8.13]	[7.31–7.65]		

**Table 6:** Distribution of overcommitment groups and irritation scale ranges within the overall sample and within the three age groups (number (%)).

Characteristics	Total (n =995)	Age Groups		
		AG I (n =332)	AG II (n =339)	AG III (n =324)
**Overcommitment**				
*OC< 18*	660	212	214	234
% of age group	66.3%	63.9%	63.1%	72.2%
% of the total number	66.3%	21.3%	21.5%	23.5%
*OC* *≥* *18*	335	120	125	90
% of age group	33.7%	36.1%	36.9%	27.8%
% of the total number	33.7%	12.1%	12.6%	9.0%
Note: *p*_χ2accordingtoPearson_ = 0.023; *p*_Goodman-and-Kruskal-Tau_ = 0.004(age-group-dependent)				
**Emotional irritation (characteristics)**				
*average*	437	147	122	168
% of age group	43.9%	44.3%	36%	51.9%
% of total number	43.9%	14.9%	12.3%	16.9%
*above average*	558	217	217	156
% of age group	56.1%	55.7%	64%	48.1%
% of total number	56.1%	18.6%	21.8%	15.7%
Note: *p*_χ2accordingtoPearson_ < 0.001; *p*_Goodman-and-Kruskal-Tau_ < 0.001(age-group-dependent)				
**Cognitive irritation (characteristics)**				
*average*	280	75	84	121
% of age group	28.1%	22.6%	24.8%	37.3%
% of total number	28.1%	7.5%	8.4%	12.2%
*above average*	715	257	255	203
% of age group	71.9%	77.4%	75.2%	62.7%
% of total number	71.9%	25.8%	25.6%	20.4%
Note: *p*_χ 2 according to Pearson_ < 0.001; *p*_Goodman-and-Kruskal-Tau_ = 0.010 (age-group-dependent)				

**Table 7:** Results from the analysis of generalized linear models.

	Cognitive Irritation		Emotional Irritation	Total Index Irritation
Corrected model	F	46.480	29.594	49.677
	*p*	<0.001	<0.001	<0.001
	η^2^	0.342	0.249	0.357
Gender	*p*	n.s.	n.s.	n.s.
	η^2^	<0.001	<0.001	<0.001
Type of employment	*p*	n.s.	n.s.	n.s.
	η^2^	0.003	<0.001	0.004
Specialist area	*p*	0.027	n.s.	n.s.
	η^2^	0.007	<0.001	0.001
Working years	*p*	n.s.	n.s.	n.s.
	η^2^	<0.001	0.003	0.001
Overcommitment groups	*p*	<0.001	<0.001	<0.001
	η^2^	0.038	0.051	0.049
Age group	*p*	n.s.	n.s.	n.s.
	η^2^	0.001	0.006	0.002

Note: *p* = significance level. n.s. = not significant; η^2^ = eta square (the assessment of the effect size was carried out according to the following scheme: η^2^ < 0.06 corresponds to a small effect; η^2^ from 0.06 to 0.14 to a medium effect; and η^2^ > 0.14 to a large effect.

**Table 8:** Nonparametric correlation according to Spearman’s rho (correlation coefficient and *p*-value).

	**[Years]**	**[Years]**	**Working** **Years**	**Irritation**			**Over** **commitment**
				**Cognitive**	**Emotional**	**Total Index**	**SC of All Items**
	**Working** **Years**	0.931 *** (<0.001)					
**Irritation**	**Cognitive**	−0.145 *** (<0.001)	−0.158 *** (<0.001)				
	**Emotional**	−0.082 * (0.010)	−0.086 ** (0.007)				
	**Total** **index**	−0.116 *** (<0.001)	−0.123 *** (<0.001)				
**Overcommitment**	**SC of all items**	−0.099 ** (0.002)	−0.104 ** (0.002)	0.618 *** (<0.001)	0.527 *** (<0.001)	0.617 *** (<0.001)	

Notes. * *p* < 0.05, ** *p* < 0.01, *** *p* < 0.001 (twi-sided significance level).

## Data Availability

No new data were created or analyzed in this study. Data sharing is not applicable to this article.

## Abbreviations

The following abbreviations are used in this manuscript:

AG	Age group
CI	Cognitive irritation
DVG	German DVG = Deutsche Veterinärmedizinische Gesellschaft = German Veterinary Medical Society
EI	Emotional irritation
HRV	Heart rate variability
IS	Irritation scale
OC	Overcommitment
TI	Total index

## References

[1] Bundestierärztekammer (2023). Statistik 2022: Tierärzteschaft in der Bundesrepublik Deutschland. Dtsch. Tierärzteblatt.

[2] Schreiber A. Wie viele Hunde, Katzen, Kleinsäuger und Vögel leben in Deutschland? Heimtierpopulation in Deutschland 2023. https://www.zzf.de/artikel/checkliste-zu-gefaehrlichem-zubehoer-fuer-heimtiere.

[3] Bundesanstalt für Landwirtschaft und Ernährung Tierhaltung. https://www.bmel-statistik.de/landwirtschaft/tierhaltung.

[4] Nienhaus A., Skudlik C., Seidler A. (2005). Work-related accidents and occupational diseases in veterinarians and their staff. Int. Arch. Occup. Environ. Health.

[5] Geuenich K. (2011). Stress im Tierärzteberuf als Gesundheitsrisiko. Dtsch. Tierärzteblatt.

[6] Platt B., Hawton K., Simkin S., Mellanby R.J. (2012). Suicidal behaviour and psychosocial problems in veterinary surgeons: A systematic review. Soc. Psychiatry Psychiatr. Epidemiol..

[7] Pohl R., Botscharow J., Böckelmann I., Thielmann B. (2022). Stress and strain among veterinarians: A scoping review. Ir. Vet. J..

[8] Thielmann B., Pohl R., Böckelmann I. (2024). Physical stress and musculoskeletal complaints of veterinarians—A narrative review. Appl. Erg..

[9] Smith M.R., Buote N.J., Sumner J.P., Freeman L.J. (2024). Variables associated with the prevalence of self-reported work-related musculoskeletal disorders in veterinary laparoscopic surgeons. Vet. Surg..

[10] Neubauer V., Gächter A., Probst T., Brühl D., Dale R., Pieh C., Humer E. (2024). Stress factors in veterinary medicine-a cross-sectional study among veterinary students and practicing vets in Austria. Front. Vet. Sci..

[11] Nett R.J., Witte T.K., Holzbauer S.M., Elchos B.L., Campagnolo E.R., Musgrave K.J., Carter K.K., Kurkjian K.M., Vanicek C.F., O’Leary D.R. (2015). Risk factors for suicide, attitudes toward mental illness, and practice-related stressors among US veterinarians. J. Am. Vet. Med. Assoc..

[12] Gardner D.H., Hini D. (2006). Work-related stress in the veterinary profession in New Zealand. N. Z. Vet. J..

[13] Batchelor C.E.M., McKeegan D.E.F. (2012). Survey of the frequency and perceived stressfulness of ethical dilemmas encountered in UK veterinary practice. Vet. Rec..

[14] Crane M.F., Phillips J.K., Karin E. (2015). Trait perfectionism strengthens the negative effects of moral stressors occurring in veterinary practice. Aust. Vet. J..

[15] Moses L., Malowney M.J., Wesley Boyd J. (2018). Ethical conflict and moral distress in veterinary practice: A survey of North American veterinarians. J. Vet. Intern. Med..

[16] Dow M.Q., Chur-Hansen A., Hamood W., Edwards S. (2019). Impact of dealing with bereaved clients on the psychological wellbeing of veterinarians. Aust. Vet. J..

[17] Bartram D.J., Yadegarfar G., Baldwin D.S. (2009). Psychosocial working conditions and work-related stressors among UK veterinary surgeons. Occup. Med..

[18] Kersebohm J., Doherr M., Becher A. (2017). Berliner und Münchener Tierärztliche Wochenschrift: Lange Arbeitszeiten, geringes Einkommen und Unzufriedenheit: Gegenüberstellung der Situation praktizierender Tiermediziner mit vergleichbaren Berufsgruppen der deutschen Bevölkerung. Lange Arbeitszeiten, geringes Einkommen und Unzufriedenheit: Gegenüberstellung der Situation praktizierender Tiermediziner mit vergleichbaren Berufsgruppen der deutschen Bevölkerung. Berl. Und Münchener Tierärztliche Wochenschr..

[19] Johnson J.V., Lipscomb J. (2006). Long working hours, occupational health and the changing nature of work organization. Am. J. Ind. Med..

[20] Bartram D.J., Baldwin D.S. (2010). Veterinary surgeons and suicide: A structured review of possible influences on increased risk. Vet. Rec..

[21] Dawson B.F.Y., Thompson N.J. (2017). The Effect of Personality on Occupational Stress in Veterinary Surgeons. J. Vet. Med. Educ..

[22] Perret J.L., Best C.O., Coe J.B., Greer A.L., Khosa D.K., Jones-Bitton A. (2020). Association of demographic, career, and lifestyle factors with resilience and association of resilience with mental health outcomes in veterinarians in Canada. J. Am. Vet. Med. Assoc..

[23] Schunter N., Glaesmer H., Lucht L., Bahramsoltani M. (2022). Depression, suicidal ideation and suicide risk in German veterinary medical students compared to the German general population. PLoS ONE.

[24] Jones-Fairnie H., Ferroni P., Silburn S., Lawrence D. (2008). Suicide in Australian veterinarians. Aust. Vet. J..

[25] Bartram D.J., Yadegarfar G., Baldwin D.S. (2009). A cross-sectional study of mental health and well-being and their associations in the UK veterinary profession. Soc. Psychiatry Psychiatr. Epidemiol..

[26] Hatch P.H., Winefield H.R., Christie B.A., Lievaart J.J. (2011). Workplace stress, mental health, and burnout of veterinarians in Australia. Aust. Vet. J..

[27] Schwerdtfeger K., Glaesmer H., Bahramsoltani M. (2020). Tierärztinnen und Tierärzte sind häufiger suizidgefährdet als andere Berufsgruppen. Dtsch. Tierärzteblatt.

[28] Platt B., Hawton K., Simkin S., Dean R., Mellanby R.J. (2012). Suicidality in the veterinary profession: Interview study of veterinarians with a history of suicidal ideation or behavior. Crisis.

[29] Dilly M., Ehrich F., Hilke J., Geuenich K. (2014). Untersuchungen zu Belastungen, Beschwerden und Ressourcen im Studium der Tiermedizin. Tierärztliche Umsch..

[30] Herbst U., Voeth M., Eidhoff A.T., Müller M., Stief S. Studierendenstress in Deutschland—Eine empirische Untersuchung. https://www.uni-heidelberg.de/md/journal/2016/10/08_projektbericht_stressstudie.pdf.

[31] Kogan L.R., Rishniw M., Hellyer P.W., Schoenfeld-Tacher R.M. (2018). Veterinarians’ experiences with near misses and adverse events. J. Am. Vet. Med. Assoc..

[32] Best C.O., Perret J.L., Hewson J., Khosa D.K., Conlon P.D., Jones-Bitton A. (2020). A survey of veterinarian mental health and resilience in Ontario, Canada. Can. Vet. J..

[33] Rohmert W., Rutenfranz J. (1975). Arbeitswissenschaftliche Beurteilung der Belastung und Beanspruchung an Unterschiedlichen Industriellen Arbeitsplätzen.

[34] Siegrist J. (1996). Adverse health effects of high-effort/low-reward conditions. J. Occup. Health Psychol..

[35] Demerouti E., Bakker A.B., Nachreiner F., Schaufeli W.B. (2001). The job demands-resources model of burnout. J. Appl. Psychol..

[36] Becker P. (2006). Gesundheit durch Bedürfnisbefriedigung.

[37] Karasek R.A., Theorell T. (1990). Healthy Work: Stress, Productivity, and the Reconstruction of Working Life.

[38] Siegrist J., Starke D., Chandola T., Godin I., Marmot M., Niedhammer I., Peter R. (2004). The measurement of effort-reward imbalance at work: European comparisons. Soc. Sci. Med..

[39] Wernecke C., Lux A., Böckelmann I., Thielmann B. (2020). Belastungsfaktoren, Overcommitment und Burnout-Risiko bei Bankangestellten unterschiedlichen Alters. Arbeitsmed Sozialmed Umweltmed.

[40] Böckelmann I., Zavgorodnii I., Litovchenko O., Kapustnyk V., Thielmann B. (2023). Berufliche Gratifikationskrisen, Verausgabungsneigung und Burnout bei ukrainischen Anästhesisten und Intensivmedizinern während der SARS-CoV-2-Pandemie. Zentralblatt Für Arbeitsmedizin Arbeitsschutz Und Ergon..

[41] Hinsch D.M., Spanier K., Radoschewski F.M., Bethge M. (2019). Associations between overcommitment, effort-reward imbalance and mental health: Findings from a longitudinal study. Int. Arch. Occup. Environ. Health.

[42] Thielmann B., Hartung J., Böckelmann I. (2022). Objective assessment of mental stress in individuals with different levels of effort reward imbalance or overcommitment using heart rate variability: A systematic review. Syst. Rev..

[43] Darius S., Hohmann C.B., Siegel L., Böckelmann I. (2022). Assessment of Psychological Stress in Kindergarten Teachers with Varying Degrees of Overcommitment. Psychiatr. Prax..

[44] Mohr G., Rigotti T., Müller A. (2007). Irritationsskala zur Erfassung arbeitsbezogener Beanspruchungen.

[45] Böckelmann I., Pohl R., Darius S., Thielmann B. (2022). Causes and consequences of psychological stress in the working life and emergency services of veterinary professionals in the Federal Republic of Germany: A protocol for a nationwide cross-sectional study [version 1; peer review: 1 approved with reservations, 1 not approved]. F1000Research.

[46] Siegrist J., Letzel S., Nowak D. (2016). Fragebogen zur Messung beruflicher Gratifikationskrisen. Handbuch der Arbeitsmedizin.

[47] Sperlich S., Arnhold-Kerri S., Siegrist J., Geyer S. (2013). The mismatch between high effort and low reward in household and family work predicts impaired health among mothers. Eur. J. Public Health.

[48] Lehr D., Koch S., Hillert A. (2010). Where is (im)balance? Necessity and construction of evaluated cut-off points for effort–reward imbalance and overcommitment. J. Occup. Organ. Psychol..

[49] Belém D., da Silva Filho C.R., Jacinto A.F., França A.B., Conterno L.O. (2021). Influence of overcommitment on the quality of life and on climacteric symptoms in nursing professionals. Rev. Gauch. Enferm..

[50] Siegrist J., Wege N., Pühlhofer F., Wahrendorf M. (2009). A short generic measure of work stress in the era of globalization: Effort-reward imbalance. Int. Arch. Occup. Environ. Health.

[51] Shimazu A., de Jonge J. (2009). Reciprocal relations between effort-reward imbalance at work and adverse health: A three-wave panel survey. Soc. Sci. Med..

[52] Rantanen J., Feldt T., Hyvönen K., Kinnunen U., Mäkikangas A. (2013). Factorial validity of the effort-reward imbalance scale: Evidence from multi-sample and three-wave follow-up studies. Int. Arch. Occup. Environ. Health.

[53] Mohr G., Rigotti T., Müller A. (2005). Irritation—ein Instrument zur Erfassung psychischer Beanspruchung im Arbeitskontext. Skalen- und Itemparameter aus 15 Studien. Z. Für Arb. Und Organ. AO.

[54] Cohen J. (1988). Statistical Power Analysis for the Behavioral Sciences.

[55] Siegrist J. (2010). Effort-reward imbalance at work and cardiovascular diseases. Int. J. Occup. Med. Environ. Health.

[56] Lunde A., Braut G.S. (2019). Overcommitment: Management in Helicopter Emergency Medical Services in Norway. Air Med. J..

[57] Bardhan R., Heaton K., Davis M., Chen P., Dickinson D.A., Lungu C.T. (2019). A Cross Sectional Study Evaluating Psychosocial Job Stress and Health Risk in Emergency Department Nurses. Int. J. Environ. Res. Public Health.

[58] Du Prel J.-B., March S., Schröder H., Peter R. (2015). Occupational gratification crisis and sickness absence in Germany: Cross-sectional results from the lidA-study. Bundesgesundheitsblatt Gesundheitsforschung Gesundheitsschutz.

[59] Tseng P.-C., Lin P.-Y., Liang W.-M., Lin W.-Y., Kuo H.-W. (2022). Effort-reward imbalance and job strain index associated with health-related quality of life for civil servants in a national survey: The mediation effect of job support and over-commitment. Int. J. Occup. Med. Environ. Health.

[60] Weigelt O., Seidel J.C., Erber L., Wendsche J., Varol Y.Z., Weiher G.M., Gierer P., Sciannimanica C., Janzen R., Syrek C.J. (2023). Too Committed to Switch Off-Capturing and Organizing the Full Range of Work-Related Rumination from Detachment to Overcommitment. Int. J. Environ. Res. Public Health.

[61] Hong Thai B.T., Nhu Trang N.T., Cam V.T., Thu Trang L., Huyen Trang P.T. (2022). Effort–reward ratio, over-commitment and burnout: A cross-sectional study among Vietnamese healthcare professionals. Cogent Psychol..

[62] Violanti J.M., Mnatsakanova A., Andrew M.E., Allison P., Gu J.K., Fekedulegn D. (2018). Effort-Reward Imbalance and Overcommitment at Work: Associations with Police Burnout. Police Q..

[63] Bahramsoltani M., Bröer S., Langforth S., Eule C., Prior A., Vogt L., Li T.-T., Schirone R., Pohl A., Jensen K.C. (2023). Outcome of Communication Training in Veterinary Studies: Influence on the Perception of the Relevance of Veterinary Competencies and Self-Assessment of Communication Skills. Animals.

[64] Berufsgenossenschaft für Gesundheitsdienst und Wohlfahrtspflege Gefährdungsbeurteilung Tiermedizin—Bgw-Online. https://www.bgw-online.de/bgw-online-de/themen/sicher-mit-system/gefaehrdungsbeurteilung/online-gefaehrdungsbeurteilungen-mit-dokumentation/gefaehrdungsbeurteilung-tiermedizin.

[65] Bundesverband Praktizierender Tierärzte e.V. Über den bpt. https://www.tieraerzteverband.de/bpt/impressum/.

[66] Hagen J.R., Weller R., Mair T.S., Kinnison T. (2020). Investigation of factors affecting recruitment and retention in the UK veterinary profession. Vet. Rec..

[67] Deutsche Veterinärmedizinische Gesellschaft Wissenschaftstransfer, Fortbildung, Förderung. https://secure.dvg.net/ueber-uns/dvg-ev/.

[68] Gralla M.S., Guendel H., Mueller A., Braehler E., Häuser W., Kruse J., Muschalla B., Rigotti T., Strauss B., Balint E.M. (2023). Validation of the irritation scale on a representative German sample: New normative data. Sci. Rep..

